# SWI/SNF Infobase—An exclusive information portal for SWI/SNF remodeling complex subunits

**DOI:** 10.1371/journal.pone.0184445

**Published:** 2017-09-29

**Authors:** Udayakumar Mani, Alagu Sankareswaran S., Arun Goutham R. N., Suma Mohan S.

**Affiliations:** School of Chemical & Biotechnology, SASTRA University, Tirumalaisamudram, Thanjavur, India; George Mason University, UNITED STATES

## Abstract

Chromatin remodeling complexes facilitate the access of condensed genomic DNA during transcription, replication, and repair, by altering the histone-DNA contacts in the nucleosome structures. SWI/SNF (SWItch/Sucrose Non-Fermentable) family of ATP dependent chromatin remodeling complexes have been documented for their tumour suppressor function. Recent studies have reported the high frequency of cancer causing mutations in this protein family. There exist multiple subunits for this complex and can form context-dependent sub-complexes. The cataloguing of individual subunits of this complex is essential for understanding their specific functions and their mechanism of action during chromatin remodeling. This would also facilitate further studies to characterize cancer causing mutations in SWI/SNF subunits. In the current study, a database containing information on the subunits of SWI/SNF-α (BRG1/BRM-Associated Factors (BAF)) and SWI/SNF-β (Polybromo-Associated BAF (PBAF)) sub classes of SWI/SNF family has been curated and catalogued. The database hosts information on 27 distinct SWI/SNF subunits from 20 organisms spanning a wide evolutionary range of eukaryotes. A non-redundant set of 522 genes coding for SWI/SNF subunits have been documented in the database. A detailed annotation on each subunit, including basic protein/gene information, protein sequence, functional domains, homologs and missense mutations of human proteins have been provided with a user-friendly graphical interface. The SWI/SNF Infobase presented here, would be a first of its kind exclusive information portal on SWI/SNF complex subunits and would be a valuable resource for the research community working on chromatin remodeling. The database is available at http://scbt.sastra.edu/swisnfdb/index.php.

## Introduction

The SWI/SNF complexes are an evolutionarily conserved family of nucleosome remodeling factors that use the energy derived from ATP hydrolysis to remodel chromatin [1]. The biochemical activities mediated by this family of remodeling complex include sliding the nucleosomes, evicting the histone octamer and removing the H2A/H2B dimers [2–4] and these chromatin rearrangements, can positively or negatively regulate the gene expression [5]. The modulation of gene expression mediated by these remodeling factors is known to influence a wide variety of cellular processes, including differentiation, proliferation and DNA repair [6]. The components of the SWI/SNF family have initially discovered in yeast three decades ago [7]. These large, multi-subunit complexes (~2MDa in size) comprises of eight or more subunits and the subunit homologs have been identified in different eukaryotes.

SWI/SNF complexes can be grouped into two major subfamilies: The first subfamily includes human BAF (BRG1-Associated Factors, SWI/SNF-α), Drosophila BAP (Brahma-associated proteins) and yeast SWI/SNF; the other one comprises of human PBAF (Polybromo-associated BAF, SWI/SNF-β), Drosophila PBAP (Polybromo-associated BAP) and yeast RSC (Remodels the structure of Chromatin) [8, 9]. The ATPase subunit is the main catalytic subunit in the complex and BAF complex of higher eukaryotes can contain either BRG1(*SMARCA4*)(gene coding the corresponding subunit in human is mentioned in the parenthesis throughout the manuscript) or BRM(*SMARCA2*) in their complex assembly, whereas PBAF complex contains only BRG1. The core subunits including ATPase subunit, BAF47 (*SMARCB1*), BAF155 (*SMARCC1*), and BAF170 (*SMARCC2*) are important for remodeling activity [10]. Various tightly associated factors known as accessory subunits such as BAF57 (*SMARCE1*), β-actin (*ACTB*), BAF53A/B (*ACTL6A/B*) and BAF60A/B/C (*SMARCD1/2/3*) contribute in targeting or regulation of the complex. The two major subfamilies BAF and PBAF, are defined by their unique signature subunits BAF250A/B (*ARID1A/ARID1B*), BAF45B/C/D (*DPF1/DPF3/DPF2*), BRD9 (*BRD9*) in BAF and BAF200 (*ARID2*), BAF45A (*PHF10*), BAF180 (*PBRM1*) and BRD7(*BRD7*) in PBAF respectively [8]. The variation in these complex specific subunits implies diverse regulatory roles. In addition, the BAF and PBAF complexes are known to regulate non-overlapping sets of target genes [11]. The presence of different DNA binding and protein-protein interaction domains in SWI/SNF subunits results in different functional subunit assemblies and subsequent nucleosome recognition in different cellular scenarios [8].

Recent cancer genome sequencing efforts have revealed that 20% of all human cancers hold mutations in gene encoding SWI/SNF subunits. Loss-of-function mutations in genes such as *ARID1A/B*, *ARID2*, *SMARCA2*, *SMARCA4*, *SMARCB1*, *SMARCE1*, *BRD7*, and *PBRM1* are prevalent in different cancer types [12, 13]. It should be noted that each subunit has its own unique functional role in the complex formation, nucleosome recognition, and remodeling. There are also cell-type specific isoforms leading to the tissue-specific complex formation that can regulate tissue-specific gene expression programs [14, 15]. However, there is a lack of comprehensive information on the different SWI/SNF functional assemblies. The vast amount of subunit information on different SWI/SNF functional assemblies is spread-out in literature. With the intention to systematically catalogue the SWI/SNF subunit information and provide a convenient access to such information, the SWI/SNF Infobase has been developed.

Though efforts have been taken to catalogue chromatin remodeling factors and epigenetic factors, no exclusive resource is available for SWI/SNF remodeling factors. CREMOFAC is a database that collates information on different families of chromatin remodeling factors from mammals and higher eukaryotes [16]. Another database called EpiFactors provides information on human epigenetic regulators and their complexes [17]. The above resources lack the detailed subunit information and recently identified subunits of BAF and PBAF. Despite the vast amount of experimental evidence on the subunits containing specific functional assemblies of SWI/SNF, there is a deficit of comprehensive information on these subunits, which necessitates an extensive SWI/SNF resource.

With this aim, we collected and curated the information available on subunits belonging to the BAF and PBAF subfamily of SWI/SNF complex from literature. The information has been extended for 20 organisms, spanning a wide evolutionary range of eukaryotes. The collected data has been made into an online database with a user-friendly interface that enables a quick and efficient search of these subunits. This SWI/SNF Infobase, a specific knowledge base containing information on BAF and PBAF subunits in 20 organisms, is dedicated to the SWI/SNF research community, who would greatly benefit from such collective resource.

## Materials and methods

### Data resources

The curated SWI/SNF subunit entries in the SWI/SNF Infobase and its assembly preferences were obtained from the published literature resource- PubMed through keyword search. This text based search builds a list of experimentally identified subunits of BAF and PBAF complexes. The annotated information for the subunits was retrieved from UniProt Knowledgebase (UniProtKB) [18]. Gene related details such as gene official symbol, full name, synonyms, location, and transcript details were collected from the NCBI Gene resource [19] and protein domain details were collected from the Pfam database [20]. Cancer associated missense mutations in human SWI/SNF subunits were obtained from the COSMIC v81 database [21]. The information about 20 species distributed in different species groups such as primates, rodents, laurasiatheria, afrotheria, other mammals, birds & reptiles, amphibia, fishes, insecta, nematode, fungi and other eukaryotes were collected. The orthologous relationship was established between distinct SWI/SNF subunits across these organisms using the domain enhanced method, DELTA-blast [22].

### Database organization and web interface

SWI/SNF Infobase was developed as an open source software system using MySQL-5.0.41 –Win32 as a back end, an open source relational database management system (RDBMS) which uses Structured Query Language (SQL), that enables efficient addition, accessing, and processing of data in the database. PHP—5.2.3 Hypertext Preprocessor was used as a front end for dynamic web page creation. The database web interface application also includes the features of jQuery and AJAX. The graphical component for displaying the subunits present in each organism upon selection was developed using Scalable Vector Graphics (SVG). The information about SWI/SNF subunits were collected from many published literature sources and to remove the redundancy of entries in the database normalization procedures were applied. Later, a database schema along with the primary key and foreign key dependencies was developed. The entries were loaded using MySQL query browser, a visual tool for creating, executing, and optimizing SQL queries.

## Results & discussion

### Summary statistics of the SWI/SNF Infobase

**Fig 1:**
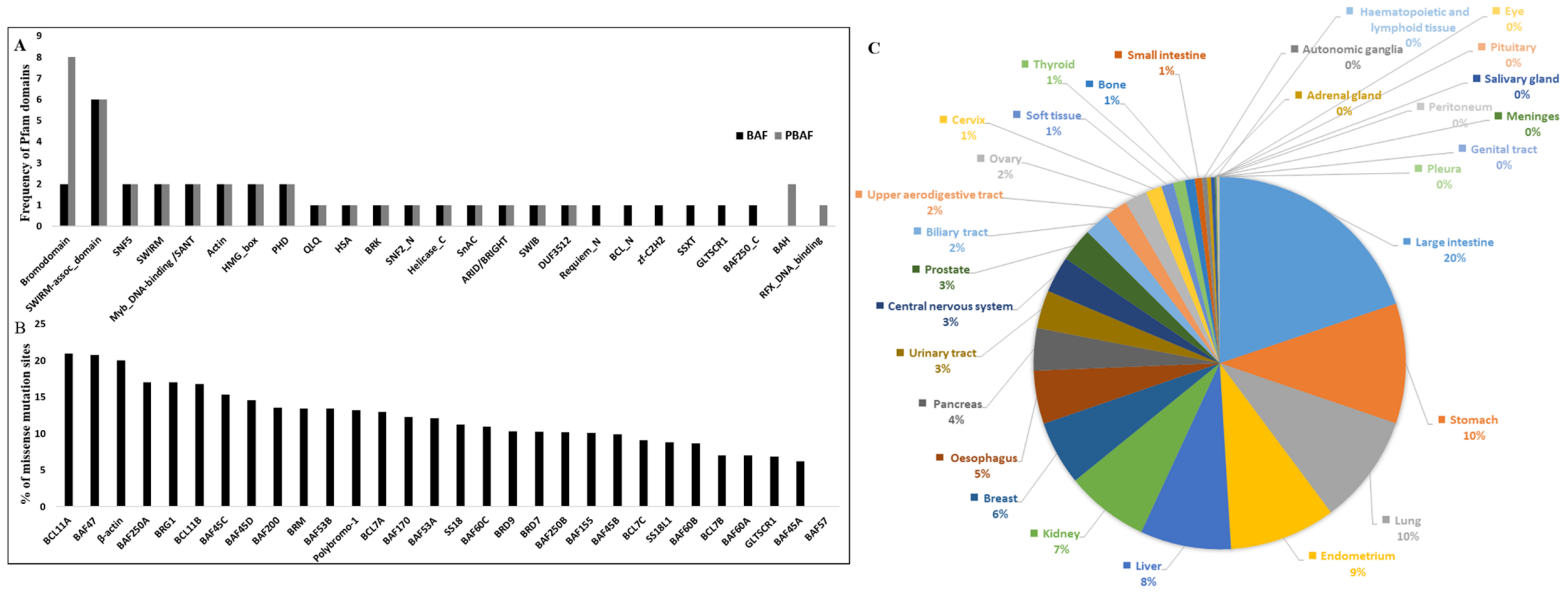
Summary statistics of Pfam domains and missense mutations in SWI/SNF subunits. (A) The frequency of Pfam domains found in the human BAF and PBAF complex assembly. (B) % of cancer associated missense mutation sites in human SWI/SNF subunits according to COSMIC database. (C) % distribution of the SWI/SNF subunit missense mutations in different primary tissue types.

The SWI/SNF Infobase currently contains information on 27 distinct subunits contributing to the formation of SWI/SNF complex (Table 1). In total, the database covers 516 SWI/SNF subunits from 20 different organisms, spanning a wide evolutionary range of eukaryotes (Table 2). Among the 516 subunits, 436 subunits form the BAF complex and 276 subunits contribute to the formation of PBAF complex. In higher eukaryotes, the BAF or PBAF subunits are encoded by 30 unique genes. There are duplications found in the genes encoding the subunits, BAF47, BAF250A, BAF155, BAF60C, β-actin and BCL7B in Zebra fish. In total, the information on 522 genes from 20 organisms coding for SWI/SNF subunits is available in the present database. Based on the included Pfam domain information, there are 23 and 19 distinct domains present in the fully functional assembly of human BAF and PBAF complexes respectively (Fig 1A).

**Table 1:** SWI/SNF subunit details from organisms, human, mouse, fruit fly, round worm, and yeast.

S. No.	Subunit name	Type of subunit	BAF (human)	PBAF (human)	BAF (mouse)	PBAF (mouse)	BAP (fruit fly)	PBAP (fruit fly	BAF (round worm)	PBAF (round worm)	SWI/SNF (yeast)	RSC (yeast)
1	ATPase	Core	BRG1/BRM	BRG1	BRG1/BRM	BRG1	Brm	Brm	SWSN-4	SWSN-4	SNF2	STH1
2	BAF47/INI1/Snr1/SNF5/SFH1	Core	BAF47	BAF47	BAF47	BAF47	Snr1	Snr1	SNFC-5	SNFC-5	SNF5	SFH1
3	ARID domain containing subunit	Signature	BAF250A/B	BAF200	BAF250A/B	BAF200	Osa	Bap170	LET-526	SWSN-7	SWI1	RSC9
4	BAF155/Moira/SWI3/RSC8	Core	BAF155	BAF155	BAF155	BAF155	Moira	Moira	SWSN-1	SWSN-1	SWI3	RSC8
5	BAF170/Moira/SWI3/RSC8	Core	BAF170	BAF170	BAF170	BAF170	Moira	Moira	SWSN-1	SWSN-1	SWI3	RSC8
6	BAF60/Bap60/SWP73/RSC6	Accessory	BAF60A/B/C	BAF60A/B/C	BAF60A/B/C	BAF60A/B/C	Bap60	Bap60	SWSN-2.1/SWSN-2.2	SWSN-2.1/SWSN-2.2	SWP73	RSC6
7	Actin	Accessory	β-actin	β-actin	β-actin	β-actin	Act5C	Act5C			ARP7	ARP7
8	Actin-related protein	Accessory	BAF53A/B	BAF53A/B	BAF53A/B	BAF53A/B	Bap55	Bap55	SWSN-6	SWSN-6	ARP9	ARP9
9	Polybromo-1/Polybromo/RSC1/2/4	PBAF specific		Polybromo-1		Polybromo-1		Polybromo		PBRM-1		RSC1/2/4
10	Bromodomain containing protein	Signature	BRD9	BRD7	BRD9	BRD7	CG7154	CG7154	SWSN-9	SWSN-9		
11	BAF45/D4/SAYP	Signature	BAF45B/C/D	BAF45A	BAF45B/C/D	BAF45A	D4	SAYP	DPFF-1	PHF-10		
12	BAF57/Bap111	Accessory	BAF57	BAF57	BAF57	BAF57	Bap111	Bap111	SWSN-3	SWSN-3		
13	BCL7	BAF specific	BCL7A/B/C		BCL7A/B/C		BCL7-like		BCL-7			
14	BCL11	BAF specific	BCL11A/B		BCL11A/B		CG9650					
15	SS18/SS18L1	BAF specific	SS18/SS18L1		SS18/SS18L1		CG10555		ZK973.9			
16	GLTSCR1	BAF specific	GLTSCR1		GLTSCR1		CG11873		MIG-38			
17	SWP82	yeast specific swi/snf									SWP82	
18	SNF6	yeast specific swi/snf									SNF6	
19	SNF11	yeast specific swi/snf									SNF11	
20	TAF14	yeast specific swi/snf									TAF14	
21	RTT102	yeast specific									RTT102	RTT102
22	RSC3	yeast specific RSC										RSC3
23	RSC30	yeast specific RSC										RSC30
24	RSC58	yeast specific RSC										RSC58
25	RSC7	yeast specific RSC										RSC7
26	HTL1	yeast specific RSC										HTL1
27	RSC14	yeast specific RSC										RSC14

**Table 2:** Number of BAF and PBAF subunits of 20 species included in SWI/SNF Infobase.

Species group	Scientific name	Common name	No of BAF subunits	No of PBAF subunits	Total no. of genes coding for BAF/PBAF subunits
Primates	*Homo sapiens*	Human	26	15	30
	*Pan troglodytes*	Chimpanzee	26	15	30
Rodents	*Mus musculus*	Mouse	26	15	30
	*Rattus norvegicus*	Rat	26	15	30
	*Cavia porcellus*	Guinea Pig	25	15	29
Laurasiatheria	*Bos taurus*	Cattle	26	15	30
	*Canis lupus familiaris*	Dog	26	15	30
	*Pteropus vampyrus*	Large flying fox	26	15	30
Afrotheria	*Loxodonta africana*	African elephant	26	15	30
Other mammals	*Monodelphis domestica*	Grey short-tailed opossum	24	13	28
Birds & Reptiles	*Gallus gallus*	Chicken	25	15	29
	*Anolis carolinensis*	Green anole	24	14	28
Amphibia	*Xenopus tropicalis*	Tropical clawed frog	26	15	30
Fishes	*Danio rerio*	Zebra fish	23	15	33
Other Eukaryotes	*Branchiostoma floridae*	Florida lancelet	14	9	14
Insecta	*Drosophila melanogaster*	Fruit fly	14	11	17
	*Anopheles gambiae*	African malaria mosquito	16	11	18
Nematode	*Caenorhabditis elegans*	Round worm	13	11	16
Other Eukaryotes	*Aplysia californica*	California sea hare	12	10	14
Fungi	*Saccharomyces cerevisiae*	Baker's yeast	12	17	26
**Total**			**436**	**276**	**522**

### SWI/SNF Infobase content

The database contains information available on core, accessory and signature subunits of BAF and PBAF complexes from 20 organisms. The subunits of SWI/SNF were initially discovered by genetic screening studies in yeast [23]. Though most of the subunits were found to be conserved between organisms, there exists variation in domain architecture between the subunits present in yeast and higher eukaryotes (data not shown). There are many yeast specific subunits identified in the SWI/SNF and RSC complexes that do not have orthologs in other organisms (listed in Table 1) [8]. In higher eukaryotes, additional subunits with specialized functions contribute to the functional diversity of the complexes. The observations based on the core, accessory and signature subunits are listed below (Table 1).

**Core subunits**
- The core subunits, ATPase(BRG1/BRM), BAF47, BAF155 and BAF170 can form the minimized complex, which can do the *in vitro* remodeling activity [10].
- The ATPase subunit does the catalytic function in the complex and in higher eukaryotes BAF complex contains either BRG1 or BRM, but PBAF contains only BRG1.
- The other core subunits BAF47, BAF155 and BAF170 are conserved between the two subclasses, BAF and PBAF.
- The subunits, BAF155 and BAF170 are differentiated only in higher eukaryotes
- Specifically, in yeast, the core subunits in SWI/SNF and RSC subfamily (which are equivalent to BAF and PBAF complexes in higher eukaryotes) are encoded by different genes.

**Accessory subunits**
- The accessory subunits, BAF60A/B/C, BAF57, β-actin and actin-related proteins (BAF53A/B) are found to be conserved between BAF and PBAF complexes
- The β-actin component is absent in lower organisms, which contain yet another actin related protein.
- In higher eukaryotes, BAF60 subunit can be either BAF60A, BAF60B or BAF60C and are known to undergo tissue specific regulation [24]
- In the case of BAF60 subunit, there are two subunits, SWP73 and RSC6 present in the SWI/SNF and RSC complexes of yeast respectively.
- BAF57 subunit does not have an ortholog in yeast.

**Signature subunits**
- The ARID domain containing signature subunit marks the difference between BAF and PBAF complexes, where BAF250A/B or BAF200 will be incorporated correspondingly.
- Another important PBAF specific subunit is Polybromo, with exception in yeast which contains three subunits RSC1/ RSC2/ RSC4.
- The other important signature subunits in the BAF and PBAF complexes, respectively are BRD9 or BRD7 and BAF45B/BAF45C/BAF45D or BAF45A. But these subunits do not have orthologs in yeast.
- Recently, there are some additional BAF specific factors identified that include, BCL7A/ BCL7B/ BCL7C, BCL11A/ BCL11B, SS18/SS18L1 and GLTSCR1 and are mainly found in higher eukaryotes [25, 26].

Generally, DNA-binding and protein-protein interaction favoring domains are present in different SWI/SNF subunits. Among the twenty-five identified functional domains, eight of them namely SWIRM, SWIRM-associated domains, SANT, SNF5, Actin, HMG-box and PHD have multiple occurrences in BAF and PBAF complexes and the frequency of these functional domains has been represented in Fig 1A. Bromodomain which recognizes acetylated lysine is the most frequently occurring domain in the PBAF complex because of the presence of Polybromo subunit containing six Bromodomains. DNA contacting domains such as High mobility group(HMG), AT-rich interaction and domain(ARID), Myb_DNA binding domain/SANT and SWIRM domain are present in both BAF as well as PBAF complexes, while the C2H2 Zinc finger is present only in BAF and RFX_DNA binding domain only in PBAF. The protein-protein interaction favoring domains like Bromodomain, QLQ domain, HSA, SnAC, SWIB/MDM2 domain, actin and PHD domain are present in both complexes. The protein-protein association favoring domains such as Bromodomain and Bromo-adjacent homology(BAH) domain show difference in frequency between BAF and PBAF complexes and this might have implications in the functional assemblies of BAF and PBAF complexes. The Requiem_N domain present in the BAF45 subunit of BAF complex functions as an adaptor between SWI/SNF and RelB [27]. The SWIRM domain of BAF155, in addition to its known DNA binding activity, is shown to interact with the BAF47 subunit [28]. The exact function of the domains such as BRK, SWIRM associated domains, DUF3512, BCL7_N and SSXT are still not known.

The percentage of cancer-associated missense mutation sites in SWI/SNF subunits is shown in Fig 1B. BCL11A, BAF47 and β-actin are the top three subunits showing more than 20% missense mutation sites. More than 5% of the residues found to be mutated in all subunits except BAF57. So far missense mutations are not identified in the BAF57 subunit. The tissue distribution of cancer associated missense mutations in SWI/SNF subunits is highlighted in Fig 1C. Among the 31 different tissue types, skin, large intestine, stomach, lung and endometrium are the top five cancer sites with SWI/SNF subunit mutations (Fig 1C).

### SWI/SNF Infobase usage

**Fig 2:**
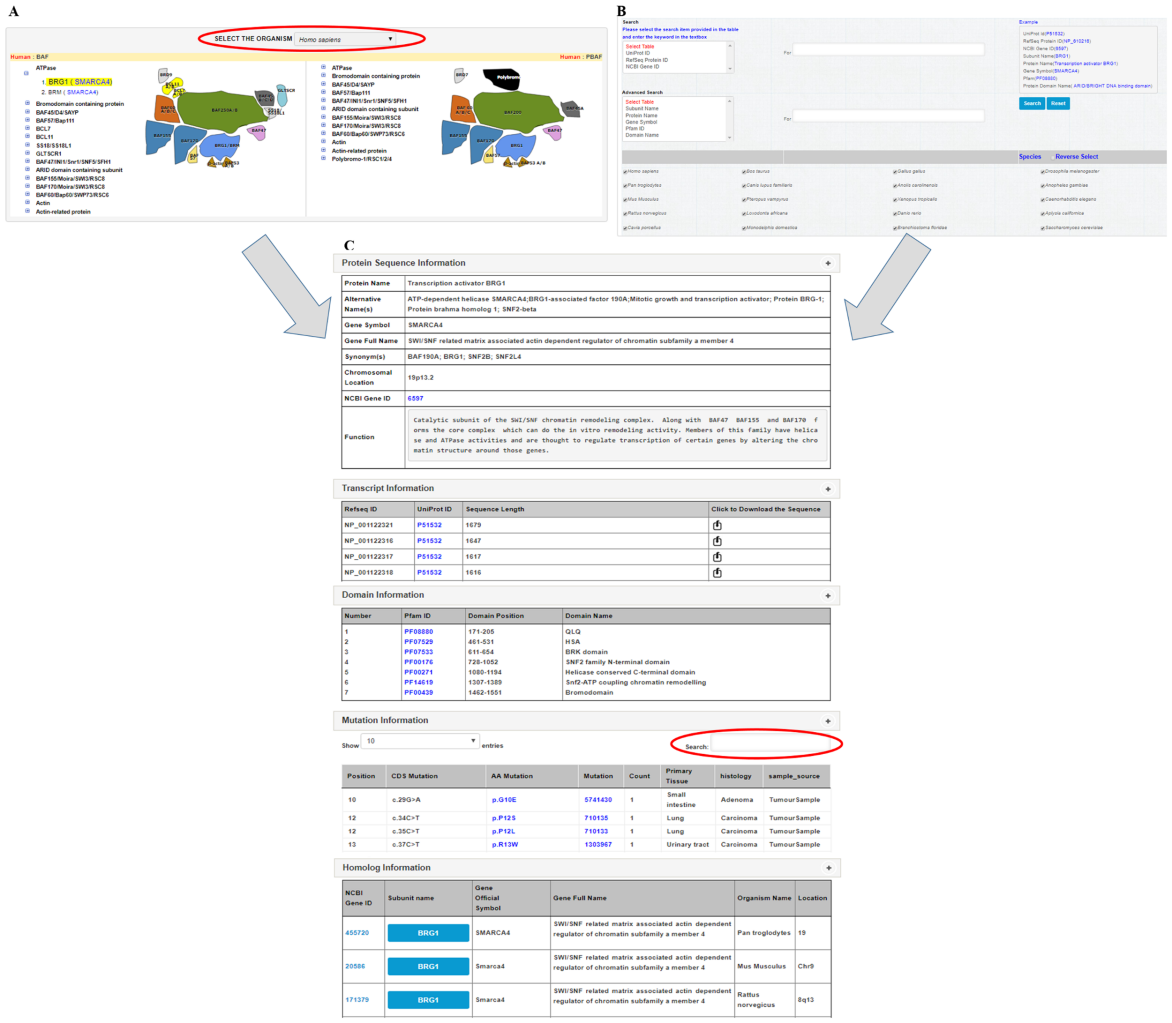
Snapshot of the SWI/SNF Infobase features. (A) The graphical interface of SWI/SNF Infobase (selection option for organism is highlighted). (B) The search page of SWI/SNF Infobase (C) Detailed subunit information provided upon the selection of the ATPase, BRG1 from *Homo sapiens* (option to list missense mutation information based on primary tissue has been highlighted).

The SWI/SNF Infobase was developed to access the SWI/SNF subunit information in a convenient way. The user can select organism name, to obtain the BAF and PBAF assembly information and subunit details of that particular organism (Fig 2). For each organism, the subunit specific information can be obtained from the subunit listing option, where each subunit expands into its sub-elements. The graphical display option provides the users with at-a-glance information on the subunits of BAF and PBAF subfamily. The cartoon representation of the subunits can also be used for navigating to specific subunit information, up on clicking. The BAF and PBAF sub-complex information is provided in the same interface to enable comparison between BAF and PBAF for each organism. Once the user selects a particular subunit, the general gene/protein information, details of transcripts, Pfam domains and homologs are provided in a new page. The subunits from human include the missense mutations from the COSMIC database. An additional option is provided in the mutation information part, where the user can select subunit mutations according to the tissue of interest. External links are provided to relevant public databases.

The search option provided in the database enables specific ID and keyword based search. The user can provide a UniProt ID, RefSeq Protein ID or NCBI Gene ID to obtain subunit information. Besides, subunit name, protein name, and gene symbol can be used for obtaining subunit information of selected organisms. The subunits with specific protein domains in different organisms can be identified using the Pfam ID or domain name based search option. Taxonomy browser can be used for listing the gene, subunit and transcript information from a specific organism. The blast option enables a homology search to the SWI/SNF Infobase and PDB database. Using the download option, the list of genes and transcripts from a specific organism can be downloaded in text format.

## Conclusions

The overall goal of SWI/SNF Infobase is to provide a comprehensive resource on the SWI/SNF complex subunits and an efficient retrieval system to facilitate further studies. The database catalogues information on 27 SWI/SNF subunits from 20 organisms from a wide evolutionary range of eukaryotes. Graphical display along with the subunit listing option of BAF and PBAF complexes in the same interface enables a comparison between BAF and PBAF subunits. The information on functional domains and subunit search option based on this provided in the database would enable a better understanding of the functional role of each subunit in the complex. The listing of homologous members would facilitate evolutionary analysis of specific subunits. Owing to the recent implications of the SWI/SNF complexes in cancers, cancer associated missense mutation details on SWI/SNF subunits provided in the database would help in identification and characterization of the tissue specific mutations in SWI/SNF subunits. The keyword or ID based search option using gene, protein and domain ID, names or symbols available in the database would aid in obtaining information on specific subunits of interest. Although there is ambiguity in the tissue specific subunits and their functional assemblies, an organized pool of existing data as furnished here, shall provide a convenient platform for exploring the SWI/SNF subunit information and enable a comparative analysis of BAF and PBAF subfamily of remodeling complexes.

## Future directions

The SWI/SNF Infobase would be continuously updated based on the availability of further information on SWI/SNF subunits. Additional features and subunit information for more organisms will be added in future releases. Further expansion will consider inclusion of additional information on subunits, gene expression, tissue specificity and genome wide binding profiles of subunits.

## Availability

The SWI/SNF Infobase has been made freely available to the research community through the web link (http://scbt.sastra.edu/swisnfdb/index.php).
